# Krukenberg tumour as the initial presentation of Peutz-Jeghers syndrome

**DOI:** 10.1093/gastro/got031

**Published:** 2013-12-19

**Authors:** Bhavith Remalayam, Santhosh Kuriakose, Prathapan Valiya Kambarath, Ramachandran Thazhath Mavali

**Affiliations:** ^1^Department of Gastroenterology, Government Medical College, Kozhikode, Kerala, India, ^2^Department of Obstetrics and Gynaecology, Government Medical College, Kozhikode, Kerala, India and ^3^Department of Surgical Gastroenterology, Government Medical College, Kozhikode, Kerala, India

**Keywords:** Krukenberg tumour, Peutz-Jeghers syndrome, signet cell adenocarcinoma

## Abstract

Peutz-Jeghers syndrome (PJS) is an hereditary syndrome characterized by gastrointestinal polyposis and mucocutaneous pigmentation. PJS patients are at increased risk of developing various cancers, especially of the gastrointestinal and gynaecological tracts. Colonic adenocarcinoma is one of the more common tumours that occur in PJS. We report a young lady presenting with a large ovarian tumour, later diagnosed to have PJS with colonic signet cell adenocarcinoma and synchronous ovarian metastasis.

## INTRODUCTION

Peutz-Jeghers syndrome (PJS) was first described by Peutz in 1921. It is characterized by mucocutanous melaninosis and gastrointestinal polyposis. Melaninosis occurs on the lips, buccal mucosa, fingers, nose, and perioral areas. Gastrointestinal polyps occur anywhere from the stomach to the colon in varying numbers. The clinical presentation of PJS is generally recurrent abdominal pain due to intussusception and anaemia due to occult or overt gastrointestinal bleeding [1]. Extra-intestinal manifestations like gall bladder polyposis and nasal polyposis rarely occur. Patients with PJS are at increased lifetime risk for various intestinal and extra-intestinal malignancies [1, 2]. Colonic and breast cancers are, respectively, the most common intestinal and extra-intestinal malignancies in PJS [2]. Female genital tract neoplasms in PJS include ovarian neoplasms from the epithelium and stromal cells, adenoma malignum of the cervix and adenocarcinoma of the endometrium. Sex cord tumour with annular tubules (SCTAT) is the most common ovarian neoplasm in PJS, which occurs in 5% of patients. Sertoli cell tumour of the ovary, mucinous and serous epithelial ovarian tumour, and ovarian mature teratoma occurs rarely in these patients [3]. We describe a case of PJS with a Krukenberg tumour as its initial presentation—later turning out to be a colonic adenocarcinoma with metastasis—which has never previously been reported in medical literature.

## CASE REPORT

**Figure 1..**
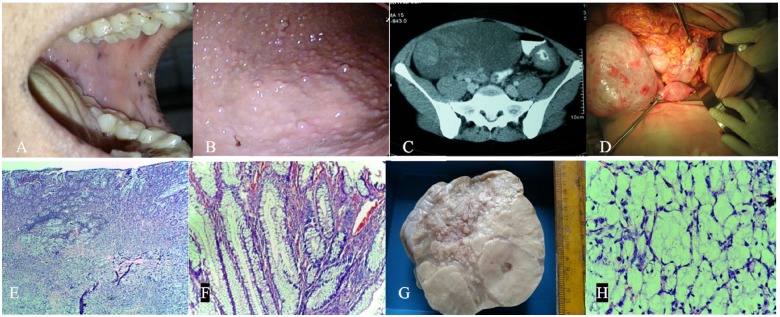
**A**: Pigmentation over lips and buccal mucosa. **B**: Multiple small polyps in the stomach. **C**: CECT showing large ovarian mass and wall thickening of descending colon. **D**: Intra operative findings showing left ovarian mass and colonic malignant lesion. **E**: Signet cell adenocarcinoma colon infiltrating serosa **F**: Microscopy of Peutz-Jeghers polyp. **G**: Ovarian cut surface showing solid mucinous areas. **H**: Mucin containing signet ring cells of ovary.

This 32-year-old female presented with history of recurrent bouts of dyspepsia over the preceding two years. There was no history of associated upper or lower gastrointestinal bleeding, abdominal pain, jaundice or menstrual irregularities. She had undergone right oophorectomy 12 years previously for an ovarian mucinous cystadenoma, the details of which could not be traced. Her brother had had history of surgery for intussusception. Physical examination revealed mucocutaneous pigmentation over the lips, buccal mucosa (Figure 1A) and fingers. Abdominal examination revealed a large, firm mass occupying both the right iliac fossa and hypogastrium. Vaginal examination revealed a 10 x 8 cm mobile mass in the right fornix. She was haemodynamically stable and other systems were clinically normal. Her investigations revealed haemoglobin 103 g/L, total leukocyte count 5.7 × 10^3^/L, platelets 362 × 10^3^/L, normal electrolytes and renal, hepatic and thyroid function tests. Tumour marker values were as follows: Beta hCG <0.10 IU/mL, AFP 1.01 IU/mL, CEA 25.25 ng/mL, and CA125 19.5 U/mL. Stool occult blood was positive.

With a high clinical suspicion of PJS, we proceeded with an upper gastrointestinal endoscopy, which revealed multiple small polyps in the stomach (Figure 1B) and large polyps in the duodenum. Colonoscopy was deferred, since the patient could not tolerate the procedure (During the procedure she developed severe pain and vasovagal syncope, at which point the procedure was terminated. Following recovery and discussion, the patient was reluctant to consent to a further colonoscopy attempt). We proceeded further with imaging studies. Her ultrasonogram revealed a large (15 × 10 × 9 cm), well encapsulated, mixed-echogenic mass with vascularity suggestive of ovarian tumour. Contrast computed tomography (CT) of the abdomen and pelvis showed a 10 × 17 × 24 cm well-defined low-density lesion in the pelvis further towards right side, with both fluid and irregular solid elements. There was circumferential wall thickening of the descending colon (Figure 1C). The proximal left ureter was dilated with distal obstruction. The right ovary was not visualized separately. A provisional diagnosis was made, of PJS with colonic malignancy and ovarian metastasis, and the decision was made to proceed with surgery. Intra-operative findings were as follows: (i) straw-coloured ascitic fluid; (ii)enlarged left ovary with a 14 × 14 cm solid mass with smooth surface (Figure 1D); (iii) absent right ovary and normal-sized uterus; (iv) solid mass lesion (5 × 5 cm) noted at the junction of descending and sigmoid colon infiltrating the serosa, with tumour infiltration to the left middle-third of the ureter, multiple omental, mesenteric, bladder, pouch of Douglas (POD) deposits; (v) a few loops of small intestine with intussusception. Surgery was performed with a multidisciplinary approach. As there was an obstructive lesion in the descending colon, extended left hemicolectomy with end-to-end anastomosis was performed. Intussuscepted loops were reduced. A left middle-third, 3 cm ureteric resection and an end-to-end anastomosis were also performed. Furthermore, total abdominal hysterectomy and left salpingo-oophorectomy, total omentectomy, bladder and POD peritonectomy were also done. Pathological examination revealed signet-cell adenocarcinoma colon infiltrating up to the serosa (Figure 1E), with Peutz-Jeghers polyps (Figure 1F) and left ovarian adenocarcinoma metastasis of signet-ring cell type. Immunohistochemistry of an ovarian specimen revealed CK 20 positivity and CK 7 negativity, suggestive of malignancy of colonic origin. The uterus and parametrium were free from tumour, whereas the peritoneum, omentum, mesentry and ureter showed tumour deposits with inferior mesenteric lymph node metastasis. A final diagnosis was made, of adenocarcinoma of the colon with Krukenberg ovarian metastasis. The patient was referred for chemotherapy. The patient is currently on a 5-fluorouracil, leucovorin and oxaliplatin [Folinic acid (leucovorin) Fluorouracil (5-FU) Oxaliplatin (Eloxatin) (FOLFOX)] regime.

## DISCUSSION

PJS is an autosomal dominant disorder caused by germ-line mutations in the STK11/LKB1 on chromosome 19p13.3 [4]. Clinical diagnosis of PJS is based on the characteristic mucocutaneous pigmentation and presence of any number of Peutz-Jeghers polyps [5]. Patients usually present in the first decade due to polyp-related complications like abdominal pain (due to obstruction and/or intussusception) and occult or overt gastrointestinal bleeding [6]. PJS patients are at increased risk of gastrointestinal and extra-intestinal cancers. Colorectal tumours are the most common malignancies of intestinal origin [2, 4]. SCTAT are unusual benign, bilateral, multifocal ovarian tumours that occur in PJS. These are very small or microscopic tumours which have morphological features intermediate between granulosa cell tumour and Sertoli cell tumour. They usually present with symptoms of hyper-oestrogenism or isosexual precocity. Although mucinous ovarian tumours in PJS occur at almost the same frequency as in the general population, they are more common than serous tumours in PJS patients, when compared with sporadically occurring cases [3].

Our patient presented in her third decade with symptoms of dyspepsia for two years. Because she lived in a backward, tribal belt of Kerala, frequent commuting to hospital was difficult and hence her symptoms were never fully evaluated at a major hospital or an institution. Ovarian mass was the striking feature on clinical examination. Characteristic pigmentation, family history of intussusception and evidence of gastrointestinal polyposis led to the diagnosis of PJS. Elevated CEA levels and CT findings were suggestive of colonic malignancy. Left ovarian mass lesion and left ureteric involvement were thought to be due to metastasis. There was diffuse peritoneal involvement and peritoneal cancer index before and after surgery was 24 and 0, respectively. As there was evidence of obstructing colonic lesion, extended left hemicolectomy was performed, followed by cytoreductive surgery. Even though the resected margins of the colon and ureter were free from tumour, the ascitic fluid cytology was positive for malignancy and microscopic disease could not be completely excluded in grossly normal areas, hence R0 resection could not be achieved. However, optimal cytoreduction to no macroscopically residual disease was performed.

Abdominal distension with dyspeptic symptoms was the presenting feature in 46% of patients with non-genital cancers metastasizing to the ovaries [7]. The most common primary site was the gastrointestinal tract, especially the stomach and colon, and CEA level was elevated in 78% of cases [7]. In patients with colonic cancer, ovarian metastasis was present in 3.4–10.3% of cases [7, 8]

Pathology was consistent with Peutz-Jeghers polyp and signet cell adenocarcinoma of the colon and ovary. Krukenberg tumour is a metastatic signet-cell adenocarcinoma of the ovary with the primary being stomach (70%), colon or the appendix. A preceding diagnosis of primary tumour is obtained in only 25% of cases. These tumours are bilateral in 80% of cases and the surface of the ovary is usually free from adhesion and deposits [9]. Our patient had a previous history of right-sided oophorectomy and the resected ovarian surface was smooth and free from tumour deposits. The cut surface was solid, with mucinous areas (Figure 1G), and microscopy showed an abundance of signet ring cells (Figure 1H). This was consistent with an earlier reported case of sigmoid colon malignancy with Krukenberg tumour [10]. Among patients having colorectal malignancy with ovarian metastasis, those who underwent optimal cytoreductive surgery had a better survival benefit than those who did not undergo the same procedure [7]. Although peritoneal carcinomatosis from colorectal cancer is considered to be a terminal condition, novel therapies including intraperitoneal chemotherapy—with or without hyperthermia (HIPEC)—have produced reports of encouraging results in open-label studies; however, this facility was not available at our institution.

## CONCLUSION

PJS usually presents with benign complications like intussusception and gastrointestinal bleeding. Colorectal malignancy is one of the most common and serious complications in these patients. As in our case, patients can rarely present in the late stages of malignancy and seek the care of the gynaecologist, rather than the gastroenterologist. Two years of delay in seeking appropriate care resulted in our patient presenting with an uncommon initial presentation of a common malignancy affecting patients with a rare underlying disorder. General practitioners need to be aware and refer patients with refractory dyspeptic symptoms—even in young individuals—for timely intervention.

**Conflict of interest:** none declared.
